# 3-[2-(5-*tert*-Butyl-1,2-oxazol-3-yl)hydrazinyl­idene]chroman-2,4-dione

**DOI:** 10.1107/S1600536813000858

**Published:** 2013-01-19

**Authors:** Ahmed Jashari, Emil Popovski, Bozhana Mikhova, Rositsa P. Nikolova, Boris L. Shivachev

**Affiliations:** a Group of Physics & Chemistry, Faculty of Natural Sciences & Mathematics, State University of Tetovo, 1200 Tetovo, Macedonia; b Institute of Chemistry, Faculty of Natural Sciences and Mathematics, Ss. Cyril and Methodius University, Arhimedova 5, 1000 Skopje, Macedonia; c Institute of Organic Chemistry with Centre of Phytochemistry, Bulgarian Academy of Sciences, Acad. G. Bonchev Str. build. 9, 1113 Sofia, Bulgaria; d Institute of Mineralogy and Crystallography, Bulgarian Academy of Sciences, Acad G. Bonchev Str. build. 107, 1113 Sofia, Bulgaria

## Abstract

In the title compound, C_16_H_15_N_3_O_4_, the dihedral angle between the chromane and isoxazole rings [r.m.s. deviations = 0.042 and 0.007 Å, respectively] is 20.33 (12)°. The mol­ecular geometry is stabilized by an intra­molecular N—H⋯O hydrogen bond. In the crystal, N—H⋯O hydrogen bonds generate chains along the *c*-axis direction. The crystal studied was a non-morohedral twin.

## Related literature

For general background to the use of coumarin derivatives in organic synthesis and as biologically active compounds see: Adavi *et al.* (2004 ▶); Shi & Zhou (2011 ▶); Toshihiro *et al.* (2005 ▶).
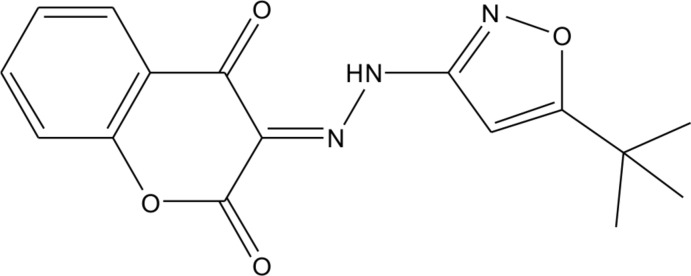


## Experimental

### 

#### Crystal data

C_16_H_15_N_3_O_4_
*M* *_r_* = 313.31
Monoclinic, 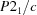

*a* = 13.431 (14) Å
*b* = 9.1803 (9) Å
*c* = 12.638 (4) Å
β = 100.49 (8)°
*V* = 1532.3 (17) Å^3^
*Z* = 4
Mo *K*α radiation
μ = 0.10 mm^−1^
*T* = 290 K
0.28 × 0.26 × 0.21 mm

#### Data collection

Agilent SuperNova (Dual, Cu at zero, Atlas) diffractometer
Absorption correction: multi-scan (*CrysAlis PRO*; Agilent, 2010 ▶) *T*_min_ = 0.584, *T*_max_ = 1.000
13050 measured reflections
3010 independent reflections
2148 reflections with *I* > 2σ(*I*)
*R*_int_ = 0.085

#### Refinement

*R*[*F* ^2^ > 2σ(*F* ^2^)] = 0.072
*wR*(*F* ^2^) = 0.181
*S* = 1.09
3010 reflections
215 parameters
H atoms treated by a mixture of independent and constrained refinement
Δρ_max_ = 0.23 e Å^−3^
Δρ_min_ = −0.16 e Å^−3^

### 

Data collection: *CrysAlis PRO* (Agilent, 2010 ▶); cell refinement: *CrysAlis PRO*; data reduction: *CrysAlis PRO*; program(s) used to solve structure: *SHELXS97* (Sheldrick, 2008 ▶); program(s) used to refine structure: *SHELXL97* (Sheldrick, 2008 ▶); molecular graphics: *ORTEP-3 for Windows* (Farrugia, 2012 ▶) and *Mercury* (Macrae *et al.*, 2006 ▶); software used to prepare material for publication: *WinGX* (Farrugia, 2012 ▶).

## Supplementary Material

Crystal structure: contains datablock(s) global, I. DOI: 10.1107/S1600536813000858/kp2443sup1.cif

Structure factors: contains datablock(s) I. DOI: 10.1107/S1600536813000858/kp2443Isup2.hkl

Supplementary material file. DOI: 10.1107/S1600536813000858/kp2443Isup3.cml

Additional supplementary materials:  crystallographic information; 3D view; checkCIF report

## Figures and Tables

**Table 1:** Hydrogen-bond geometry (Å, °)

*D*—H⋯*A*	*D*—H	H⋯*A*	*D*⋯*A*	*D*—H⋯*A*
N2—H2⋯O2	0.89 (4)	1.86 (4)	2.581 (3)	137 (4)
N2—H2⋯O9^i^	0.89 (4)	2.70 (4)	3.249 (4)	121 (3)

Symmetry code: (i) 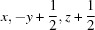.

**Fig. 1.**
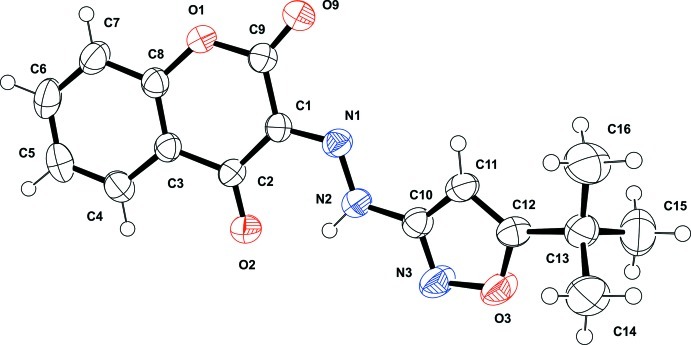
View of the structure and the atom-numbering scheme of the title compound showing 50% probability displacement ellipsoids. H atoms are shown as small spheres of arbitrary radii.

**Fig. 2.**
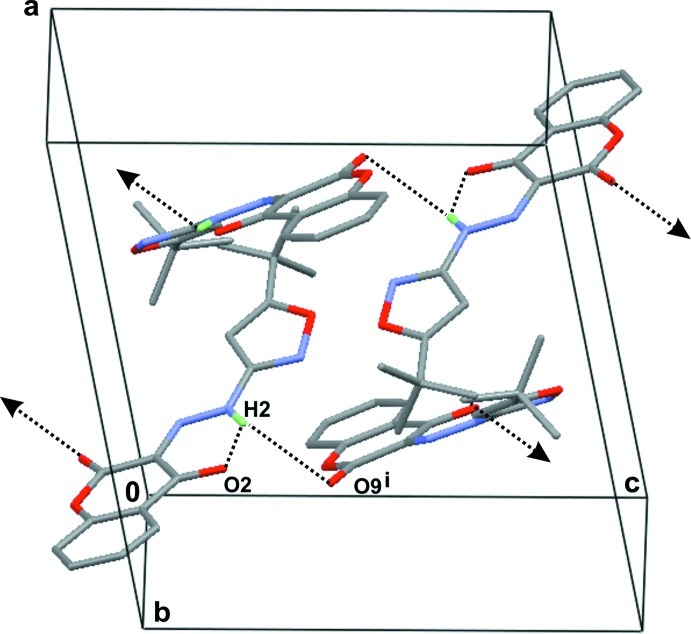
The packing arrangement of the molecules in the unit cell, showing the hydrogen-bonding interactions as dotted lines. [symmetry code: (i) x, 1/2 - y, 1/2 + z].

## supplementary crystallographic information

### Comment

Synthesis of novel coumarin derivatives is rapid ongoing process in the world due to the remarkable broad spectrum of pharmacological activities, especially anticancer (Toshihiro, *et al.*, 2005; Shi & Zhou, 2011; Adavi, *et al.*, 2004). Our interest is to develop novel coumarin compounds as efficient antitumor agents. Herein, we report the crystal structure of the title compound.

The title compound (Fig. 1) possesses two distinct functional groups: 3-iminochroman-2,4-dione, and 5-(*tert*-butyl)isoxazole. The chromane and isoxazole moieties are nearly planar (with respective r.m.s. of 0.042 and 0.007 Å).

The interplanar angle between the chromane and isoxazole is 20.33 (12)°. The molecular geometry is stabilised by the intramolecular hydrogen bond N2—H2···O2 (Table 1). In the crystal structure the molecules are connected by the N—H···O hydrogen bond forming chains along *c* (Fig. 2).

### Experimental

Suitable crystals of the title compound were obtained by slow evaporation from ethanol at room temperature.

### Refinement

All H atoms bonded to C were placed in idealized positions (C—H_aromatic_ = 0.93 Å and *C*—H_methyl_ = 0.96 Å) while the N2 hydrogen atom coordinates were located from the difference Fourier map. All H atoms were constrained to ride on their parent atoms, with *U*_iso_(H) = 1.2*U*_eq_(C or N) and 1.5*U*_eq_(C_methyl_). The observed non-morohedral twinning affected quality of data.

### Crystal data

**Table:**

C_16_H_15_N_3_O_4_	*F*(000) = 656
*M**_r_* = 313.31	*D*_x_ = 1.358 Mg m^−^^3^
Monoclinic, *P*2_1_/*c*	Melting point: 419 K
Hall symbol: -P 2ybc	Mo *K*α radiation, λ = 0.7107 Å
*a* = 13.431 (14) Å	Cell parameters from 4079 reflections
*b* = 9.1803 (9) Å	θ = 3.0–29.4°
*c* = 12.638 (4) Å	µ = 0.10 mm^−^^1^
β = 100.49 (8)°	*T* = 290 K
*V* = 1532.3 (17) Å^3^	Prism, yellow
*Z* = 4	0.28 × 0.26 × 0.21 mm

### Data collection

**Table:**

Agilent SuperNova (Dual, Cu at zero, Atlas) diffractometer	3010 independent reflections
Radiation source: SuperNova (Mo) X-ray Source	2148 reflections with *I* > 2σ(*I*)
Mirror monochromator	*R*_int_ = 0.085
Detector resolution: 10.3974 pixels mm^-1^	θ_max_ = 29.4°, θ_min_ = 3.0°
ω scans	*h* = −16→18
Absorption correction: multi-scan (*CrysAlis PRO*; Agilent, 2010)	*k* = −11→12
*T*_min_ = 0.584, *T*_max_ = 1.000	*l* = −15→16
13050 measured reflections	

### Refinement

**Table:**

Refinement on *F*^2^	Primary atom site location: structure-invariant direct methods
Least-squares matrix: full	Secondary atom site location: difference Fourier map
*R*[*F*^2^ > 2σ(*F*^2^)] = 0.072	Hydrogen site location: inferred from neighbouring sites
*wR*(*F*^2^) = 0.181	H atoms treated by a mixture of independent and constrained refinement
*S* = 1.09	*w* = 1/[σ^2^(*F*_o_^2^) + (0.0555*P*)^2^ + 1.4334*P*] where *P* = (*F*_o_^2^ + 2*F*_c_^2^)/3
3010 reflections	(Δ/σ)_max_ < 0.001
215 parameters	Δρ_max_ = 0.23 e Å^−^^3^
0 restraints	Δρ_min_ = −0.16 e Å^−^^3^

### Special details

**Table:**

Experimental. CrysAlisPro, Agilent Technologies, Version 1.171.36.20 (release 27-06-2012 CrysAlis171 .NET) (compiled Jul 11 2012,15:38:31) FTIR (KBr): 1377 cm^-1^ (CH_3_*δ*_s_), 1467.5 cm^-1^ (N=N), 1740 cm^-1^ (C=O). NMR atom numbering is according to IUPAC^13^C NMR (62.9 MHz, DMSO-d6): *δ* 28.2 (CH_3_), 32.1 (C *t-but*), 91.2 (NCCH), 117.4 (C8 of the coumarin ring), 120.4 (C4a of the coumarin ring), 124.9 (C6 of the coumarin ring), 125.7 (C3 of the coumarin ring), 126.8 (C5 of the coumarin ring), 137.0 (C7 of the coumarin ring), 154.1(C8a of the coumarin ring), 157.6 (C2 of the coumarin ring), 162.4 (NCCH), 178.3 (C4 of the coumarin ring), 182.8 (NC).^1^H NMR (250 MHz, DMSO-d_6_): *δ* 1.35 (s, 9*H*, *t-but*.), 6.57 (s,1*H* of the isoxazol ring), 7.37 (dd, *J* = 8.0, 1.5 Hz, 1*H*, H8 of the coumarin ring), 7.39 (ddd, *J* = 8.0, 8.0, 1.5 Hz, *1*H, H6 of the coumarin ring), 7.80 (ddd, *J* = 8.0, 8.0, 1.5 Hz, 1H, C7 of the coumarin ring), 8.00 (dd,*1*H, dd, *J* = 8.0, 1.5 Hz, C5 of the coumarin ring). Elemental analysis: C, 61.34; H, 4.83; N, 13.41. TOF MS ES+: m/e: 336[*M*+Na]^+^.
Geometry. All e.s.d.'s (except the e.s.d. in the dihedral angle between two l.s. planes) are estimated using the full covariance matrix. The cell e.s.d.'s are taken into account individually in the estimation of e.s.d.'s in distances, angles and torsion angles; correlations between e.s.d.'s in cell parameters are only used when they are defined by crystal symmetry. An approximate (isotropic) treatment of cell e.s.d.'s is used for estimating e.s.d.'s involving l.s. planes.
Refinement. Refinement of *F*^2^ against ALL reflections. The weighted *R*-factor *wR* and goodness of fit *S* are based on *F*^2^, conventional *R*-factors *R* are based on *F*, with *F* set to zero for negative *F*^2^. The threshold expression of *F*^2^ > σ(*F*^2^) is used only for calculating *R*-factors(gt) *etc*. and is not relevant to the choice of reflections for refinement. *R*-factors based on *F*^2^ are statistically about twice as large as those based on *F*, and *R*- factors based on ALL data will be even larger.

### Fractional atomic coordinates and isotropic or equivalent isotropic displacement parameters (Å^2^)

**Table:**

	*x*	*y*	*z*	*U*_iso_*/*U*_eq_	
C1	0.1628 (2)	0.3116 (3)	0.0511 (2)	0.0362 (6)	
C2	0.1605 (2)	0.4578 (3)	0.0958 (2)	0.0384 (6)	
C3	0.1281 (2)	0.5746 (3)	0.0182 (2)	0.0369 (6)	
C4	0.1307 (2)	0.7207 (3)	0.0498 (3)	0.0467 (7)	
H4	0.1511	0.7448	0.1219	0.056*	
C5	0.1035 (3)	0.8282 (3)	−0.0247 (3)	0.0571 (9)	
H5	0.1074	0.9254	−0.0035	0.068*	
C6	0.0701 (3)	0.7922 (4)	−0.1321 (3)	0.0580 (9)	
H6	0.0509	0.8657	−0.1823	0.070*	
C7	0.0651 (3)	0.6488 (4)	−0.1651 (3)	0.0508 (8)	
H7	0.0421	0.6250	−0.2369	0.061*	
C8	0.0948 (2)	0.5405 (3)	−0.0893 (2)	0.0389 (6)	
C9	0.1240 (2)	0.2823 (3)	−0.0636 (2)	0.0402 (6)	
C10	0.2869 (2)	0.0916 (3)	0.2637 (2)	0.0408 (6)	
C11	0.3172 (2)	−0.0401 (3)	0.2229 (2)	0.0421 (7)	
H11	0.3060	−0.0719	0.1519	0.051*	
C12	0.3667 (2)	−0.1101 (3)	0.3116 (2)	0.0432 (7)	
C13	0.4211 (3)	−0.2535 (3)	0.3317 (3)	0.0494 (8)	
C14	0.5276 (3)	−0.2268 (4)	0.3960 (3)	0.0741 (12)	
H14A	0.5637	−0.1627	0.3563	0.111*	
H14B	0.5630	−0.3178	0.4080	0.111*	
H14C	0.5229	−0.1831	0.4639	0.111*	
C15	0.3615 (4)	−0.3513 (5)	0.3961 (4)	0.0790 (12)	
H15A	0.3550	−0.3036	0.4621	0.118*	
H15B	0.3967	−0.4419	0.4119	0.118*	
H15C	0.2954	−0.3696	0.3546	0.118*	
C16	0.4268 (4)	−0.3246 (5)	0.2235 (3)	0.0769 (12)	
H16A	0.3597	−0.3363	0.1827	0.115*	
H16B	0.4586	−0.4183	0.2356	0.115*	
H16C	0.4657	−0.2640	0.1844	0.115*	
N1	0.20237 (19)	0.1960 (3)	0.10479 (18)	0.0390 (5)	
N2	0.2352 (2)	0.2092 (3)	0.20786 (19)	0.0429 (6)	
N3	0.3136 (2)	0.1048 (3)	0.3673 (2)	0.0549 (7)	
O1	0.08744 (16)	0.3990 (2)	−0.12688 (14)	0.0433 (5)	
O2	0.18801 (19)	0.4820 (2)	0.19257 (15)	0.0562 (6)	
O3	0.36552 (18)	−0.0258 (3)	0.39890 (16)	0.0558 (6)	
O9	0.1194 (2)	0.1661 (2)	−0.10662 (17)	0.0594 (7)	
H2	0.231 (3)	0.296 (5)	0.238 (3)	0.076 (13)*	

### Atomic displacement parameters (Å^2^)

**Table:**

	*U*^11^	*U*^22^	*U*^33^	*U*^12^	*U*^13^	*U*^23^
C1	0.0388 (15)	0.0349 (14)	0.0353 (14)	−0.0036 (11)	0.0082 (11)	0.0027 (10)
C2	0.0438 (16)	0.0395 (15)	0.0313 (13)	−0.0033 (12)	0.0057 (11)	0.0029 (11)
C3	0.0358 (14)	0.0379 (14)	0.0372 (13)	−0.0038 (11)	0.0071 (11)	0.0043 (11)
C4	0.0489 (18)	0.0393 (16)	0.0515 (17)	−0.0027 (13)	0.0079 (14)	−0.0030 (13)
C5	0.064 (2)	0.0336 (16)	0.076 (2)	−0.0014 (14)	0.0165 (19)	0.0061 (14)
C6	0.055 (2)	0.0499 (19)	0.067 (2)	0.0043 (15)	0.0054 (17)	0.0252 (16)
C7	0.0509 (19)	0.0549 (19)	0.0435 (16)	0.0002 (15)	0.0000 (14)	0.0131 (14)
C8	0.0362 (14)	0.0378 (14)	0.0420 (14)	−0.0032 (11)	0.0047 (12)	0.0046 (11)
C9	0.0434 (16)	0.0399 (15)	0.0374 (14)	−0.0035 (12)	0.0072 (12)	0.0014 (12)
C10	0.0366 (14)	0.0439 (16)	0.0408 (14)	0.0000 (12)	0.0043 (11)	0.0022 (12)
C11	0.0395 (15)	0.0507 (17)	0.0341 (13)	−0.0037 (13)	0.0011 (11)	0.0016 (12)
C12	0.0389 (16)	0.0498 (18)	0.0394 (15)	0.0017 (12)	0.0034 (12)	−0.0018 (12)
C13	0.0468 (18)	0.0475 (18)	0.0500 (17)	0.0080 (14)	−0.0015 (14)	−0.0013 (13)
C14	0.048 (2)	0.070 (2)	0.091 (3)	0.0149 (18)	−0.0216 (19)	−0.004 (2)
C15	0.088 (3)	0.060 (2)	0.087 (3)	0.002 (2)	0.011 (2)	0.016 (2)
C16	0.082 (3)	0.072 (3)	0.073 (3)	0.025 (2)	0.006 (2)	−0.018 (2)
N1	0.0403 (13)	0.0437 (13)	0.0329 (11)	−0.0042 (10)	0.0064 (10)	0.0017 (9)
N2	0.0500 (15)	0.0410 (14)	0.0355 (12)	0.0030 (11)	0.0022 (11)	0.0009 (10)
N3	0.0614 (18)	0.0571 (17)	0.0414 (14)	0.0205 (13)	−0.0029 (12)	−0.0005 (12)
O1	0.0517 (12)	0.0418 (11)	0.0336 (10)	−0.0026 (9)	0.0000 (9)	0.0015 (8)
O2	0.0862 (18)	0.0425 (12)	0.0360 (11)	−0.0027 (11)	0.0010 (11)	−0.0033 (9)
O3	0.0627 (15)	0.0603 (14)	0.0393 (11)	0.0232 (11)	−0.0041 (10)	0.0009 (10)
O9	0.0917 (19)	0.0402 (12)	0.0429 (12)	−0.0031 (11)	0.0028 (12)	−0.0056 (9)

### Geometric parameters (Å, º)

**Table:**

C1—N1	1.319 (4)	C10—N2	1.403 (4)
C1—C2	1.459 (4)	C11—C12	1.356 (4)
C1—C9	1.473 (4)	C11—H11	0.9300
C2—O2	1.231 (3)	C12—O3	1.350 (4)
C2—C3	1.465 (4)	C12—C13	1.505 (4)
C3—C8	1.386 (4)	C13—C16	1.530 (5)
C3—C4	1.397 (4)	C13—C14	1.531 (5)
C4—C5	1.367 (5)	C13—C15	1.531 (6)
C4—H4	0.9300	C14—H14A	0.9600
C5—C6	1.390 (5)	C14—H14B	0.9600
C5—H5	0.9300	C14—H14C	0.9600
C6—C7	1.379 (5)	C15—H15A	0.9600
C6—H6	0.9300	C15—H15B	0.9600
C7—C8	1.388 (4)	C15—H15C	0.9600
C7—H7	0.9300	C16—H16A	0.9600
C8—O1	1.381 (3)	C16—H16B	0.9600
C9—O9	1.194 (3)	C16—H16C	0.9600
C9—O1	1.373 (3)	N1—N2	1.303 (3)
C10—N3	1.299 (4)	N2—H2	0.89 (4)
C10—C11	1.404 (4)	N3—O3	1.408 (3)
			
N1—C1—C2	125.2 (2)	O3—C12—C13	116.1 (2)
N1—C1—C9	113.4 (2)	C11—C12—C13	134.8 (3)
C2—C1—C9	121.4 (2)	C12—C13—C16	108.9 (3)
O2—C2—C1	121.8 (2)	C12—C13—C14	109.1 (3)
O2—C2—C3	122.1 (3)	C16—C13—C14	110.4 (3)
C1—C2—C3	116.0 (2)	C12—C13—C15	108.6 (3)
C8—C3—C4	118.9 (3)	C16—C13—C15	109.9 (3)
C8—C3—C2	119.6 (2)	C14—C13—C15	109.9 (3)
C4—C3—C2	121.5 (3)	C13—C14—H14A	109.5
C5—C4—C3	120.5 (3)	C13—C14—H14B	109.5
C5—C4—H4	119.8	H14A—C14—H14B	109.5
C3—C4—H4	119.8	C13—C14—H14C	109.5
C4—C5—C6	119.9 (3)	H14A—C14—H14C	109.5
C4—C5—H5	120.0	H14B—C14—H14C	109.5
C6—C5—H5	120.0	C13—C15—H15A	109.5
C7—C6—C5	120.8 (3)	C13—C15—H15B	109.5
C7—C6—H6	119.6	H15A—C15—H15B	109.5
C5—C6—H6	119.6	C13—C15—H15C	109.5
C6—C7—C8	118.9 (3)	H15A—C15—H15C	109.5
C6—C7—H7	120.5	H15B—C15—H15C	109.5
C8—C7—H7	120.5	C13—C16—H16A	109.5
O1—C8—C3	122.7 (2)	C13—C16—H16B	109.5
O1—C8—C7	116.3 (3)	H16A—C16—H16B	109.5
C3—C8—C7	121.0 (3)	C13—C16—H16C	109.5
O9—C9—O1	116.7 (3)	H16A—C16—H16C	109.5
O9—C9—C1	126.2 (3)	H16B—C16—H16C	109.5
O1—C9—C1	117.1 (2)	N2—N1—C1	118.1 (2)
N3—C10—C11	113.9 (3)	N1—N2—C10	118.5 (3)
N3—C10—N2	117.1 (3)	N1—N2—H2	118 (3)
C11—C10—N2	129.0 (3)	C10—N2—H2	123 (3)
C12—C11—C10	103.6 (2)	C10—N3—O3	103.7 (2)
C12—C11—H11	128.2	C9—O1—C8	122.6 (2)
C10—C11—H11	128.2	C12—O3—N3	109.6 (2)
O3—C12—C11	109.1 (3)		
			
N1—C1—C2—O2	−7.1 (5)	N2—C10—C11—C12	177.9 (3)
C9—C1—C2—O2	176.2 (3)	C10—C11—C12—O3	0.2 (3)
N1—C1—C2—C3	170.3 (3)	C10—C11—C12—C13	−178.8 (4)
C9—C1—C2—C3	−6.4 (4)	O3—C12—C13—C16	−172.7 (3)
O2—C2—C3—C8	−177.9 (3)	C11—C12—C13—C16	6.2 (5)
C1—C2—C3—C8	4.8 (4)	O3—C12—C13—C14	−52.1 (4)
O2—C2—C3—C4	2.7 (4)	C11—C12—C13—C14	126.8 (4)
C1—C2—C3—C4	−174.7 (3)	O3—C12—C13—C15	67.7 (4)
C8—C3—C4—C5	−1.8 (5)	C11—C12—C13—C15	−113.4 (4)
C2—C3—C4—C5	177.7 (3)	C2—C1—N1—N2	5.7 (4)
C3—C4—C5—C6	1.9 (5)	C9—C1—N1—N2	−177.3 (3)
C4—C5—C6—C7	−0.7 (6)	C1—N1—N2—C10	−173.5 (3)
C5—C6—C7—C8	−0.6 (5)	N3—C10—N2—N1	−176.1 (3)
C4—C3—C8—O1	−178.3 (3)	C11—C10—N2—N1	5.8 (5)
C2—C3—C8—O1	2.2 (4)	C11—C10—N3—O3	0.2 (4)
C4—C3—C8—C7	0.5 (4)	N2—C10—N3—O3	−178.2 (2)
C2—C3—C8—C7	−179.0 (3)	O9—C9—O1—C8	−175.2 (3)
C6—C7—C8—O1	179.6 (3)	C1—C9—O1—C8	6.2 (4)
C6—C7—C8—C3	0.7 (5)	C3—C8—O1—C9	−8.2 (4)
N1—C1—C9—O9	5.7 (5)	C7—C8—O1—C9	173.0 (3)
C2—C1—C9—O9	−177.3 (3)	C11—C12—O3—N3	−0.1 (4)
N1—C1—C9—O1	−175.9 (2)	C13—C12—O3—N3	179.1 (3)
C2—C1—C9—O1	1.2 (4)	C10—N3—O3—C12	−0.1 (4)
N3—C10—C11—C12	−0.2 (4)		

### Hydrogen-bond geometry (Å, º)

**Table:**

*D*—H···*A*	*D*—H	H···*A*	*D*···*A*	*D*—H···*A*
N2—H2···O2	0.89 (4)	1.86 (4)	2.581 (3)	137 (4)
N2—H2···O9^i^	0.89 (4)	2.70 (4)	3.249 (4)	121 (3)

Symmetry code: (i) *x*, −*y*+1/2, *z*+1/2.
